# How are patterned movements stored in working memory?

**DOI:** 10.3389/fpsyg.2023.1074520

**Published:** 2023-02-17

**Authors:** Congchong Li, Wenqing Tian, Yang He, Chaoxian Wang, Xianyang Wang, Xiang Xu, Lifeng Bai, Ting Xue, Yang Liao, Tao Xu, Xufeng Liu, Shengjun Wu

**Affiliations:** ^1^Department of Military Medical Psychology, Air Force Medical University, Xi’an, China; ^2^Air Force Bureau of Trainee Pilot Selection, Nanjing Central Division, Nanjing, China; ^3^Department of Social Sciences, Aviation University of Air Force, Changchun, China; ^4^Air Force Medical Center, Air Force Medical University, Beijing, China; ^5^Secondary Air Force Healthcare Center for Special Services, Hangzhou, China

**Keywords:** patterned movement, visual working memory, spatial working memory, working memory capacity, motion animation

## Abstract

**Introduction:**

In this study, the change detection paradigm was used to study the working memory of patterned movements and the relationship of this type of memory with the visuospatial sketchpad in three experiments.

**Methods:**

Experiment 1 measured participants’ working memory capacity for patterned movements and explored the influence of stimulus type with indicators such as response time and accuracy rate. Experiments 2 and 3 explored the relationship between patterned movements and the visual and spatial subsystems, respectively.

**Results:**

The results of Experiment 1 indicated that individuals can store 3–4 patterned movements in working memory; however, a change in stimulus format or an increase in memory load may decrease the speed and efficiency of working memory processing. The results of Experiment 2 showed that working memory and visual working memory are independent when processing patterned movements. The results of Experiment 3 showed that the working memory of patterned movements was affected by spatial working memory.

**Discussion:**

Changes in stimulus type and memory load exerted different effects on the working memory capacity of participants. These results provide behavioral evidence that the storage of patterned movement information is independent of the visual subsystem but requires the spatial subsystem of the visuospatial sketchpad.

## 1. Introduction

Working memory preserves and manipulates limited visual information in real time, which is essential for cognitive functions such as visual perception, auditory perception, speech processing, planning, and reasoning (Baddeley, 2003, 2012; Zhang and Luck, 2008). The basic unit of visual information is the object, which has two basic properties, namely, features (such as shape, size, and color) and spatiotemporal information (such as position, distance, the direction of movement, and speed of movement; Smith and Jonides, 1999; Klauer and Zhao, 2004; Darling et al., 2006; Jackson et al., 2011; Mutluturk and Boduroglu, 2014; Zhao et al., 2020). Objects in our environment move and change, resulting in different perceptual events (Puce and Perrett, 2003; Masumoto et al., 2006; Blake and Shiffrar, 2007; Blakemore, 2008; Pavlova, 2012; He et al., 2019). Recent studies have revealed that the processing and memory of actions may be linked to mental disorders and normal variations in social interactions. For example, difficulties in perceiving actions are related to multiple mental disorders, including schizophrenia (Thakkar et al., 2014) and autism (von Hofsten and Rosander, 2012; Pokorny et al., 2015). When observing human behavior, we detect the movement of body parts (e.g., limbs) over time and resolve those movements into discrete units of action (Morey et al., 2019). Observed actions must be stored in working memory to preserve information after the action is perceived (Li et al., 2013; Isik et al., 2017; Thornton, 2018). This limb movement information is stored in the brain along with the featural and spatiotemporal information; this information is used to produce subsequent behavioral responses (Russ et al., 2003; Masumoto et al., 2006; Peelen et al., 2006; Gao et al., 2016).

The concept of working memory has been widely recognized by researchers since Baddeley introduced it in 1974 (Baddeley and Buchanan, 1975). According to Baddeley’s multiple component model, working memory mainly consists of four subsystems, which are: (1) the phonological loop, which is responsible for storing and maintaining speech or sound information; (2) the visuospatial sketchpad, which is used to save and process visual and spatial information; (3) the central executive system, a resource-limited system responsible for the functioning of the entire working memory system, including the normal operation of the previous two subsystems and the interaction of each subsystem of working memory with other cognitive modules, such as long-term memory; and (4) the episodic buffer, which plays an important role in the process of information reconstruction and binding as it interacts with other subsystems and long-term memory, representing the information in each system by multidimensional coding (Baddeley, 2012).

However, at present, research on the multi-component model of working memory has mainly focused on the storage of object features, spatiotemporal information, and speech information as well as the associations among these types of information (Luck and Vogel, 1997; Vogel et al., 2001; Son et al., 2020; Zhao et al., 2020). These studies ignore the issue of storage of limb movement information. Limb movement is part of an individual’s daily life. People need to identify and categorize various limb movements every day. Exploring the storage of this patterned movement information may provide an in-depth understanding of how individuals store various types of information and how different types of information are transcoded and interact (Buszard et al., 2017; Kimura et al., 2017; Xing et al., 2021; Lin et al., 2022). Baddeley (2012) speculated that a multi-component model of working memory should also contain tactile and kinesthetic information and suggested that specific subsystems may store tactile and kinesthetic information (Baddeley, 2012). Therefore, the theoretical issues posed by the storage of patterned movement information in working memory have yet to be addressed.

In the field of perceptual and working memory research, Smyth et al. (1988) classified body movements into spatial position movement and patterned movement according to the purpose of the movement (Smyth et al., 1988). Spatial position movement is the movement to move toward the position where the target is located in the external space. Patterned movement is a movement to change body configuration. The patterned movement has several characteristics. First, it can only move with specific body parts, such as ballet poses, for instance, you cannot shrug your shoulders by moving your feet. Second, patterned movement requires a high degree of precision for movement. Third, body parts and configurations are very important for patterned movement (Smyth and Pendleton, 1989). Due to the differences in the storage mechanisms of the two types of limb movement information in working memory. Therefore, it is common for researchers to explore the two types of limb movements, spatial position movement and body pattern movement, separately. However, there are still many controversies regarding the research on the working memory capacity of body pattern movements and their storage mechanisms. In this paper, we focus on body pattern movements in human limb movements.

Smyth et al. (1988) used live demonstrations of movements as experimental stimuli to investigate the working memory capacity for patterned movements. The authors asked participants to observe and memorize a series of body or hand movements that were presented to them face-to-face by the experimenter. Participants were asked to repeat the body movements they remembered either immediately after the demonstrated movements in the correct order twice in a row, they were considered to have memorized the movement sequence. In these studies, 4–5 patterned movements could be stored in working memory in the absence of concurrent interfering tasks.

Some researchers have suggested that patterned movement information is stored independently of the visual and spatial subsystems. Shen et al. (2014) used point-light display (PLD) sequences to explore whether working memory capacity and its storage buffers were independent of general visual information (e.g., color, shape, and spatial location of objects) in PLD sequences (Shen et al., 2014; Ding et al., 2015; Gao et al., 2015). The PLD sequences depicted biological motion through a series of light points (e.g., 12 light points) placed on the joints of a human body. Shen et al. (2014) found that the working memory capacity for these PLD sequences was 3–4 movements. In addition, when the PLD sequences were memorized along with information on their color and spatial position, memory performance did not decrease when compared to that when solely memorizing PLD sequences, indicating that the PLD sequences were stored in separate working memory buffers from information on their color and spatial position.

However, other researchers have argued that patterned movements and visual information are independent, despite sharing the same storage space. This theory is supported by numerous behavioral experiments and neuroscience findings. Neuroimaging studies have shown that early in visual processing, information about vision and motion is processed by different neural substrates. For example, viewing human images activates occipitotemporal areas (Downing P. E. et al., 2001; Downing P. et al., 2001; Downing et al., 2006), whereas viewing body movements activates mirror neurons in the prefrontal cortex that are not sensitive to the identity of the actor (Ruby and Decety, 2001). Cai et al. (2018) compared the neural basis of working memory for patterned movements with visual and spatial working memory. Using 3D animations, the authors assessed the working memory capacity of subjects for patterned movements, spatial information, and visual information. The authors found that brain regions involved in the retention of spatial information [i.e., the superior parietal lobule (SPL) and the superior frontal gyrus (SFG)] were also involved in the retention of patterned movements and those brain regions associated with object surface features (i.e., the fusiform gyrus) were not activated during the working memory task involving patterned movements. These findings suggest that the neural basis of working memory for patterned movements is separate from that of visual working memory. The maintenance of patterned movements, although requiring the involvement of the spatial subsystem, also activates the mesotemporal visual area (MT), a brain region independent of the visual and spatial subsystems. Thus, the results of Cai et al. (2018) provide evidence that patterned movement information is stored independently of the visual subsystem of the visuospatial sketchpad. However, this information is dependent on the spatial subsystem, suggesting that the MT is likely to be the neural basis of patterned movement information (Cai et al., 2018).

In summary, recent findings all indicated that working memory capacity is limited. However, the perceived working memory capacity differs among research (Gu et al., 2019; Guo et al., 2020). It depends heavily on the presentation of specific cues in the visual input, such as the spatial location of the stimulus, the time interval between stimulus presentations, and the physical attributes of the actor (e.g., type and color of clothing, facial expression, and gender). Whether different stimulus types alter the results remains unclear. Therefore, it is important to explore the effect of stimulus type on the working memory of patterned movement. In addition, the storage of patterned movement information in the generally accepted multi-component model of working memory is unclear. Although brain imaging studies have revealed the neural basis for the storage of patterned movement information, less evidence is available regarding the relationship between patterned movement information and the visuospatial sketchpad. Moreover, direct behavioral evidence is lacking, and more empirical studies are needed to validate the findings of the above studies. Based on this theoretical and empirical evidence, the present study used a change detection paradigm to investigate the effects of three different stimulus forms on the working memory of patterned movements. To ensure complete consistency in the accuracy and fluency of the pattern movements presented to the subjects and to improve the ecological validity of the experiment, the stimuli presented on the screen will be used as the stimulus source in this experiment. Additionally, the association between the storage of patterned movement information and the visuospatial sketchpad was assessed through behavioral experiments.

## 2. Experiment 1: Effect of different stimulus on the working memory of patterned movement

### 2.1. Methods

#### 2.1.1. Participants

The sample size was estimated using G*Power 3.1 software with the following parameters: α = 0.05, 1-β = 0.90, and a moderate effect size of 0.25. The minimum sample size was calculated to be 38 individuals. Considering the sample size will be affected by participant dropout and experimental fitting, the sample size of this study was expanded based on the previous estimation. 48 participants were recruited, all male, with a mean age of 19.63 ± 0.67 years. All participants were right-handed with normal or corrected-to-normal visual acuity, and none of the participants had previously participated in similar experiments. Informed consent was obtained from all participants before the experiment. Afterward, participants received 100 renminbi (RMB) as remuneration.

#### 2.1.2. Experimental design

This experiment had a 3 (stimulus type: PLD, 3D animation, or video) × 4 (set size (n): 2, 3, 4, or 5) within-subjects design. All participants were tested on all three stimulus types in a random and counterbalanced order. In order to control the effects of test order and individual differences, the participants were randomly divided into 6 groups. The stimuli were presented in a different order for each group of participants (Silverman, 2010; Wang et al., 2022). Each of the set sizes (2, 3, 4, or 5) and stimulus types (PLD, 3D animation, or video) contained 36 trials. Thus, 144 trials in total were randomly presented in the experiment. The experiment was divided into 3 blocks, with a 2-min break between each block, and the whole experiment took 25 min in total. Before the formal experiment, each participant completed at least 10 practice trials to ensure that they understood the experimental procedure. Additionally, to avoid introducing practice effects that could alter the experimental results, different stimulus formats and memory tasks were spaced 2 weeks apart.

#### 2.1.3. Experimental procedure

**Figure 1:**
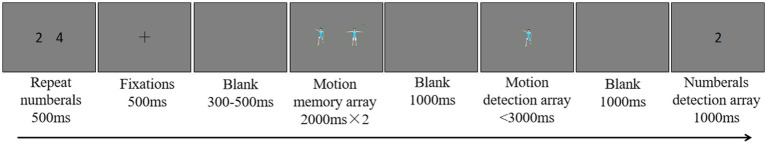
Experimental procedure. A representative trial for a set size of 2 and with 3D-animation stimuli is presented.

After each trial started, 2 numbers appeared in the center of the screen for 500 ms. The participants were asked to repeat the two numbers aloud throughout the trial. Thus, the task prohibited the use of verbal coding in the memory task. Previous studies have found that a digit recall task, compared to other interfering tasks, is better at preventing participants from utilizing verbal encoding and does not consume visual working memory resources. Subsequently, a fixation point appeared in the center of the screen for 300 ms, reminding the participants that they would be performing a motion memory task. After a blank screen was presented for 150–350 ms, a variable number of motion stimuli appeared on the screen for the participants to memorize. The presentation time was determined by the set size to be memorized (e.g., 2 motion stimuli were presented for 2 × 2,000 ms). After a blank screen was presented for 1,000 ms, a motion stimulus was presented in the center of the screen, and the participant was asked to determine whether that stimulus was one of the memorized stimuli. If the motion stimulus or number was not one of the memorized items, the participant was instructed to press the F key. If the action stimulus was one of the memorized items, the participant was instructed to press the J key. The study used accuracy as the main indicator, so correct responses were emphasized. Both the stimulus and number in the probe term had a 50% probability of changing. In the change condition, the probe stimulus was not one of the memorized items. The experimental procedure is shown in Figure 1.

#### 2.1.4. Experimental materials

The PLD stimuli used in this study were selected from the Vanrie and Verfaillie (2004) database of seven actions: hacking, crawling, biking, drinking, jumping, saluting, and walking (Vanrie and Verfaillie, 2004). Each PLD sequence consisted of 13 light points, and each motion consisted of 30 animated frames played twice in a loop. Thus, the duration of each PLD sequence was 2 s.

The 3D animation stimuli were computer-generated with MAYA 2015 3D modeling and animation software to create a human model that acted. Adobe after effect was used for post-production and rendering. The height of the model was 1.80 meters, the model wore a light blue (RGB: 67, 180, 200) shirt and light gray (RGB: 225, 219, 223) shorts, and the image was set to a brightness of 1. The light source was global + parallel illumination. The lens faced the model at a horizontal distance of 3.60 m, and 1.60 m above the ground. The final animation had a gray (RGB: 128, 128, 128) background, a duration of 2 s, a resolution of 240 × 240 pixels, and a frame rate of 30 fps. In the first 5 frames, the model maintained the same position as the starting position; in the 30th frame, the model reached the middle pause position (at which point the action was repeated); in the 56th frame, the model returned to the starting position; and in the last 5 frames, the model maintained the same position as the starting position.

The video stimuli were recorded with a video camera and consisted of a gymnastics teacher demonstrating the same movements as shown in the 3D animation. Each movement consisted of 30 frames and was looped 2 times. Thus, the duration of each stimulus video was 2 s. The demonstrator maintained the starting position for the first 5 frames, reached the middle pause position at the 30th frame (at which point the action was repeated), returned to the starting position in the 56th frame, and remained in the starting position for the last 5 frames.

#### 2.1.5. Stimulus presentation

The experimental stimuli were presented on a 27” LED monitor with a resolution of 1,920 × 1,080 pixels and a refresh rate of 60 Hz. During the stimulus presentation, the participant’s eyes were approximately 65 cm from the screen, and the motion stimuli were presented on the screen with a size of approximately 6.1° × 6.1°. In each trial, two to five motion stimuli were randomly presented in each trial, distributed on an invisible circle with a radius of approximately 7.6°, centered on the screen, and had a gray (RGB: 128, 128, 128) background. Stimulus presentation and timing control were managed using E-Prime 3.0 software.

#### 2.1.6. Statistical analysis

Among the participant data for correct responses, those with response times less than 200 ms and outside of 3 standard deviations were removed (less than 1% of participant data were finally removed). The participant’s data were also excluded when their correct digit detection item was outside the overall 3-standard deviation range. No participant’s data were removed for this reason. In all experimental conditions, the numerical judgment data were not analyzed because the accuracy rate exceeded 90%, and these data were not the main focus of this study.

The Cowan formula was used to calculate the working memory capacity for patterned movements, K=S×(H−F). H (the hit rate) refers to the probability of a participant correctly indicating when a change occurs, FA (the false alarm rate) refers to the probability that a participant incorrectly indicates a change when one did not occur, and N (the set size) indicates the memory load (Cowan, 2001, 2008).

To increase the accuracy of estimates of working memory capacity, we used an iterative approach in memory tasks with different set sizes (Alvarez and Cavanagh, 2008). In this method, the working memory capacity for patterned movements (K) was first averaged over all set sizes. Starting from the smallest set size, set sizes with K values smaller than the average K value were excluded, and the K values of the remaining set sizes were averaged again. This process was repeated until a stable mean K value was achieved.

The data obtained from this experiment were analyzed by SPSS 22.0. The data were first tested for normality using the S–W test, and the results indicated that the data of this study conformed to the normal distribution (p>0.05). A two-factor repeated-measures ANOVA was conducted to test, the working memory capacity in the change perception task. The Mauchly method was used to test the sphericity hypothesis. If the sphericity hypothesis was violated (*p* < 0.05), the Greenhouse–Geisser method was used to correct the degrees of freedom (Joanne and Rogan, 1979). The Benjamini–Hochberg (B-H) method was used to correct the *p* value to reduce the increased false discovery rate of multiple comparisons (Williams et al., 1999), at a significance level of *p* < 0.05.

### 2.2. Results

**Figure 2:**
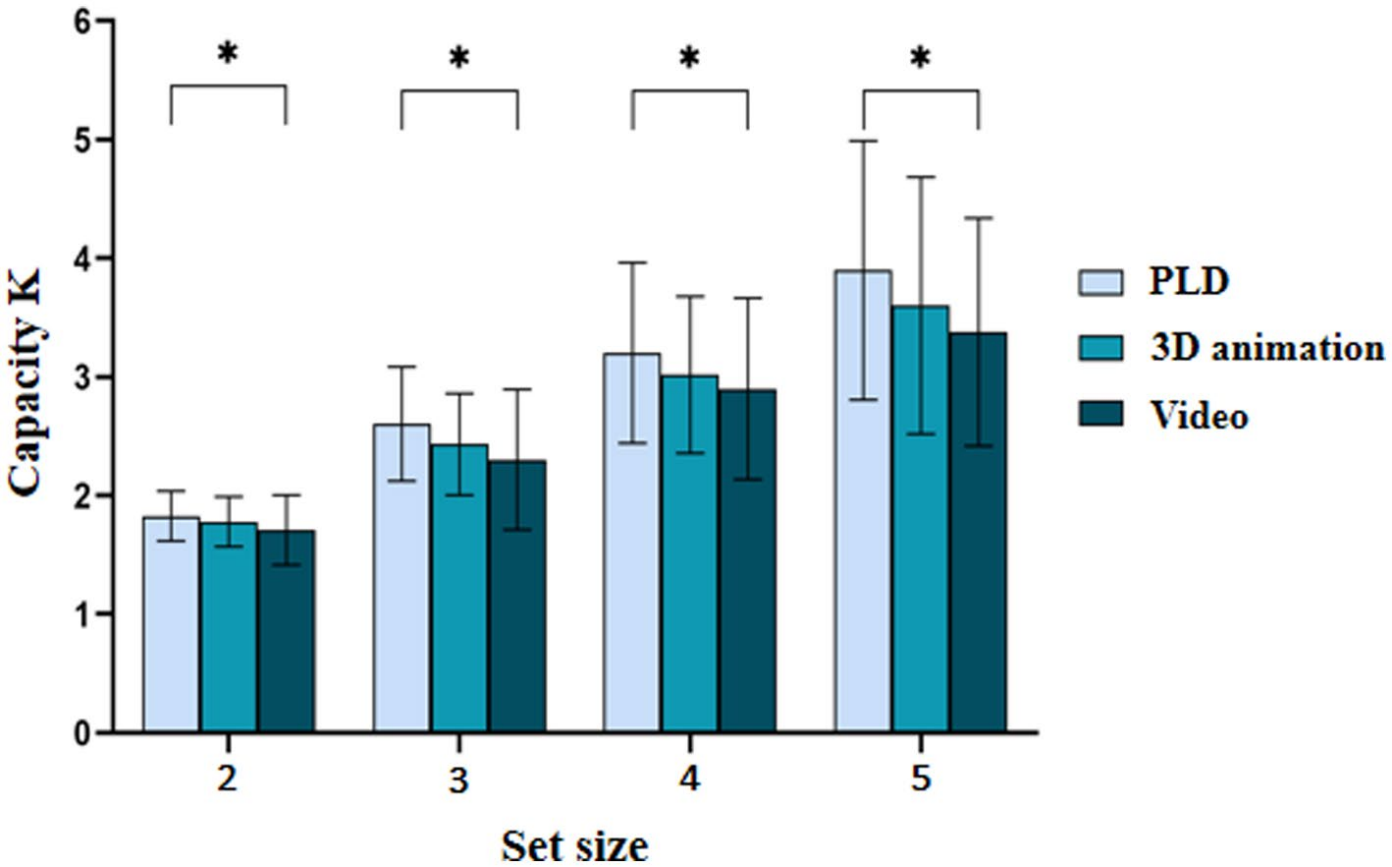
Working memory capacity for patterned movements with different stimulus types. *p < 0.05; **p < 0.01; ***p < 0.001.

**Figure 3:**
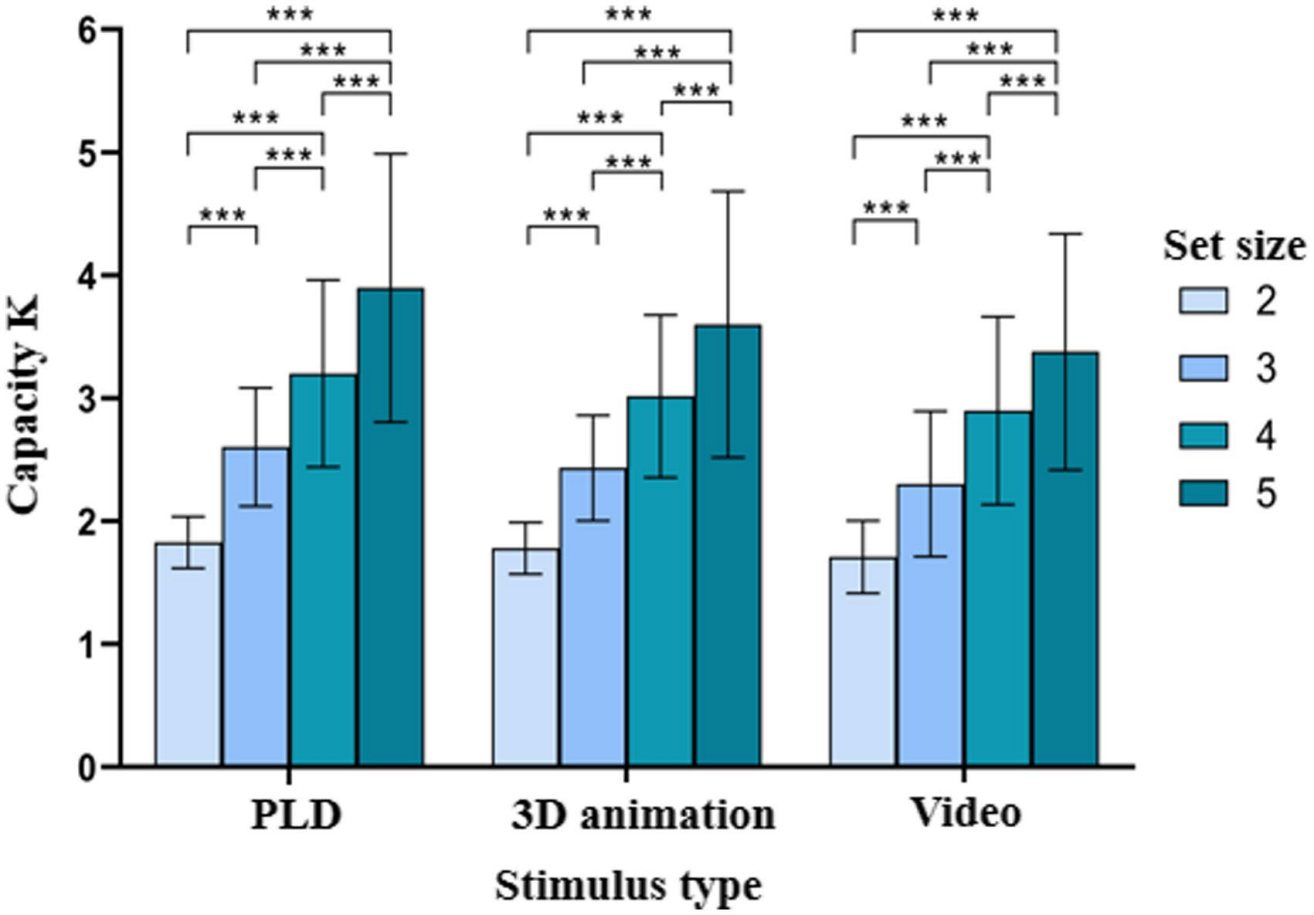
Comparison of the working memory capacity for patterned movements according to set size and stimulus type. *p < 0.05; **p < 0.01; ***p < 0.001.

Based on the results of the sphericity test was significant, Mauchly W = 0.241, *p* < 0.001. The Greenhouse–Geisser method was used to correct degrees of freedom. The repeated-measures ANOVA indicated that (1) the main effect of stimulus type was significant $\left[F\left(2,94\right)=9.288,\;p<0.001,\;\eta_{p}^{2}=0.165\right]$; *post hoc* tests revealed that K was significantly greater for the PLD sequences than for the video stimuli (p=0.006), (2) the main effect of set size was significant $\left[F\left(3,141\right)=229.774,\;p<0.001,\;\eta_{p}^{2}=0.824\right]$; *post hoc* tests showed that K significantly differed among the different set sizes (p<0.001), and (3) the interaction between stimulus type and set size was not significant $\left[F\left(6,282\right)=1.467,p=0.232,\eta_{p}^{2}=0.030\right]$. The results of Experiment 1 are shown in Table 1; Figures 2, 3.

**Table 1:** Mean accuracy, reaction time, and working memory capacity (K) values for all conditions in Experiment 1.

Stimulus format	Set size	Accuracy	Reaction time (ms)	Working memory capacity (K)
Point-light display	2	0.95 ± 0.06	1045.72 ± 226.00	1.83 ± 0.21
	3	0.93 ± 0.08	1131.17 ± 221.01	2.60 ± 0.48
	4	0.90 ± 0.10	1218.84 ± 241.50	3.20 ± 0.76
	5	0.89 ± 0.11	1258.95 ± 243.18	3.90 ± 1.09
3D animation	2	0.94 ± 0.05	1353.76 ± 219.49	1.78 ± 0.21
	3	0.90 ± 0.07	1449.94 ± 234.22	2.43 ± 0.43
	4	0.87 ± 0.09	1512.26 ± 263.51	3.02 ± 0.66
	5	0.86 ± 0.11	1546.56 ± 242.97	3.60 ± 1.08
Video	2	0.92 ± 0.08	1609.44 ± 195.32	1.71 ± 0.29
	3	0.88 ± 0.10	1702.23 ± 205.82	2.30 ± 0.59
	4	0.86 ± 0.10	1744.48 ± 198.26	2.90 ± 0.76
	5	0.83 ± 0.10	1801.48 ± 208.36	3.38 ± 0.96

## 3. Experiment 2: Relationship between patterned movement and visual subsystems

Two behavioral experiments (Experiment 2a and Experiment 2b) were designed to explore the relationship between the working memory capacity for patterned movements and visual working memory. We calculated the ratio of single to mixed types of stimuli in the working memory capacity of participants. This approach was taken to examine the relationship between the storage of pattern movements and the visual subsystem.

### 3.1. Experiment 2a: Storage of patterned movement information and color information

#### 3.1.1. Methods

##### 3.1.1.1. Participants

A total of 32 students (13 females, aged 18–23 years, mean age 20.36 ± 0.97 years) participated in Experiment 2. Participants were recruited from a military medical university, volunteered to participate in the experiment, and were given monetary compensation after the experiment. All participants had normal or corrected-to-normal vision (acuity and color perception). Participants were unaware of the purpose of the study and signed an informed consent form before the experiment. To ensure adequate statistical power, this study used G*Power 3.1 to estimate the required sample size for the experiment. Based on previous relevant behavioral studies, a minimum sample size of 18 participants was calculated as necessary to obtain a large effect size (Cohen’s d = 0.40) with an α level of 0.05 and a statistical efficacy of 90%. Considering the sample size will be affected by subject shedding and experimental fitting, the sample size of this study was expanded based on the previous estimation. Therefore, the 32 participants recruited for this study ensured that there was sufficient statistical power.

##### 3.1.1.2. Experimental design

Experiment 2a used a 2 (stimulus type: motion or color) by 2 (task type: single or mixed stimulus type) within-subjects design. In half of the trials, the probe item was a motion stimulus. In the other half, the probe item was a color stimulus. In half of the trials, the task consisted of a single stimulus type. In the other half of the trials, the task consisted of mixed stimulus types. Experiment 2a and all other experiments used a single-item change detection task (a type of change detection paradigm). In this task, during the detection phase, only one item (the probe item) was presented in the center of the screen, and participants were asked to determine whether the item had been previously presented as a memorized item. In half of the trials, the probe item was one of the memorized items, and in the other half, the probe item was not one of the memorized items. The novel probe items were randomly selected from the remaining stimuli. Each participant underwent 32 trials in each condition, preceded by 10 practice trials, for a total of 128 trials per participant. The order of the conditions was counterbalanced among participants.

##### 3.1.1.3. Experimental procedure

**Figure 4:**
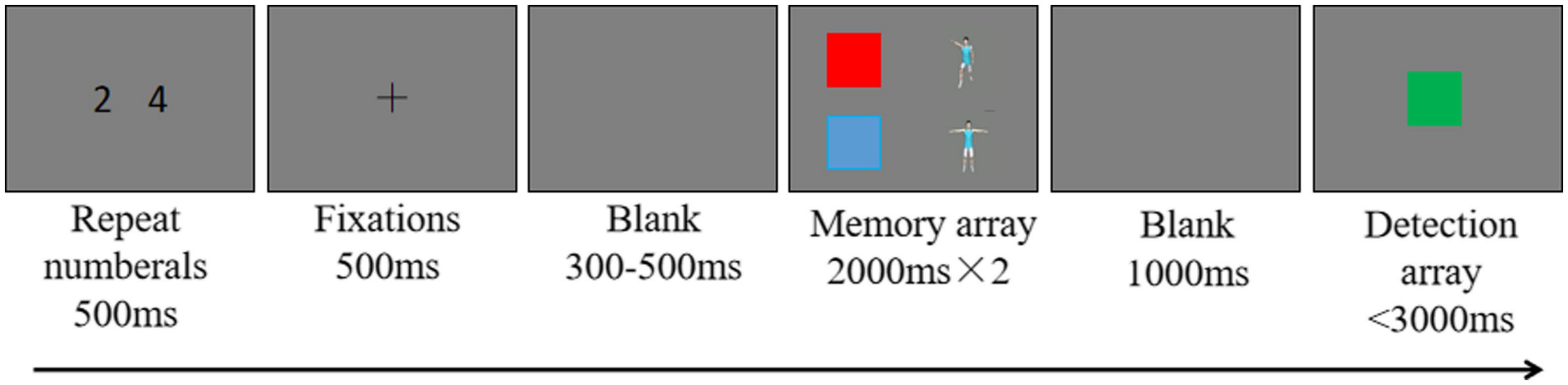
Experimental procedure. Representative images are shown for the mixed task (two stimulus types: color and motion).

The single-session procedure of Experiment 2a is shown in Figure 4. Each trial involved a 500-ms sequence of articulatory suppression, a task that suppresses the use of verbal coding in memory tasks. In this articulatory suppression task, two digits randomly selected from 1 to 9 were presented on either side of the center of the screen and the participant was instructed to repeat the two digits aloud until the end of the allotted time. A fixation cross was then presented for 500 ms and remained on the screen throughout the experiment. Next, the memorized items were presented in two rectangular areas for 2,000 ms or 4,000 ms (depending on the number of memorized items; the aim was to ensure that each item had the same encoding time: 2,000 ms/item). After the memorized items disappeared, a blank screen was presented for 1,000 ms, and then a single probe item was presented in the center of the screen for 3,000 ms or until the participant responded. Participants were instructed to determine whether the probe item was one of the memorized items. If so, they pressed “J,” and if not, they pressed “F.” Participants were further instructed to favor accuracy over speed to the extent possible when responding.

##### 3.1.1.4. Experimental stimuli

The motion stimuli were selected from the 3D animation stimuli in Experiment 1, and the color stimuli were solid blocks of the seven following colors: red (RGB: 236, 26, 35), orange (RGB: 255, 125, 39), yellow (RGB: 253, 241, 0), green (RGB: 34, 176, 76), blue (RGB: 0, 161, 231), black (RGB: 0, 0, 0), and purple (RGB: 162, 72, 162).

##### 3.1.1.5. Stimulus presentation

Stimulus presentation followed the same methods as in Experiment 1.

##### 3.1.1.6. Statistical analysis

First, the data with response times exceeding 3 standard deviations from the mean were excluded. Using the same Cowan formula as in Experiment 1 (Cowan, 2001), the working memory capacity of the participants in the different conditions was calculated. The data were tested for normality using the S–W test. The test results indicated that the data of this study conformed to the normal distribution (p>0.05). Next, we conducted a two-factor repeated-measures ANOVA on the working memory capacity K for stimulus type (motion and color) × task type (single type and mixed type).

The results of Experiment 1 showed that up to 3.22 body pattern motions could be stored in working memory. Therefore, when participants memorized 2 patterned movements, 62% of the space in working memory used to store patterned movements was already used up. The value of 62% (2/3.22 × 100%) can be used as a criterion to examine whether patterned movements and colors share the same storage buffer. If patterned movements and colors share the same storage buffer, the ratio of working memory capacity K when memorizing two stimuli to that when memorizing four stimuli would be close to 62%. If these two stimuli do not share the same memory buffer, the mean of this ratio would be lower than 62% and closer to 50%. Assuming that patterned movements and colors share the same storage buffer, remembering two patterned movements and two colors is equivalent to maintaining four patterned movements in working memory. Then we calculated the total number of stimuli kept in WM in the single-type condition of patterned movements (K-single) and the mixed-type and time condition (K-mixed) for each participant. The ratio was then computed as K-single/K-mixed for each participant. This was used to examine the relationship between the storage of patterned movement information and the visual subsystem (Shen et al., 2014).

#### 3.1.2. Results

**Figure 5:**
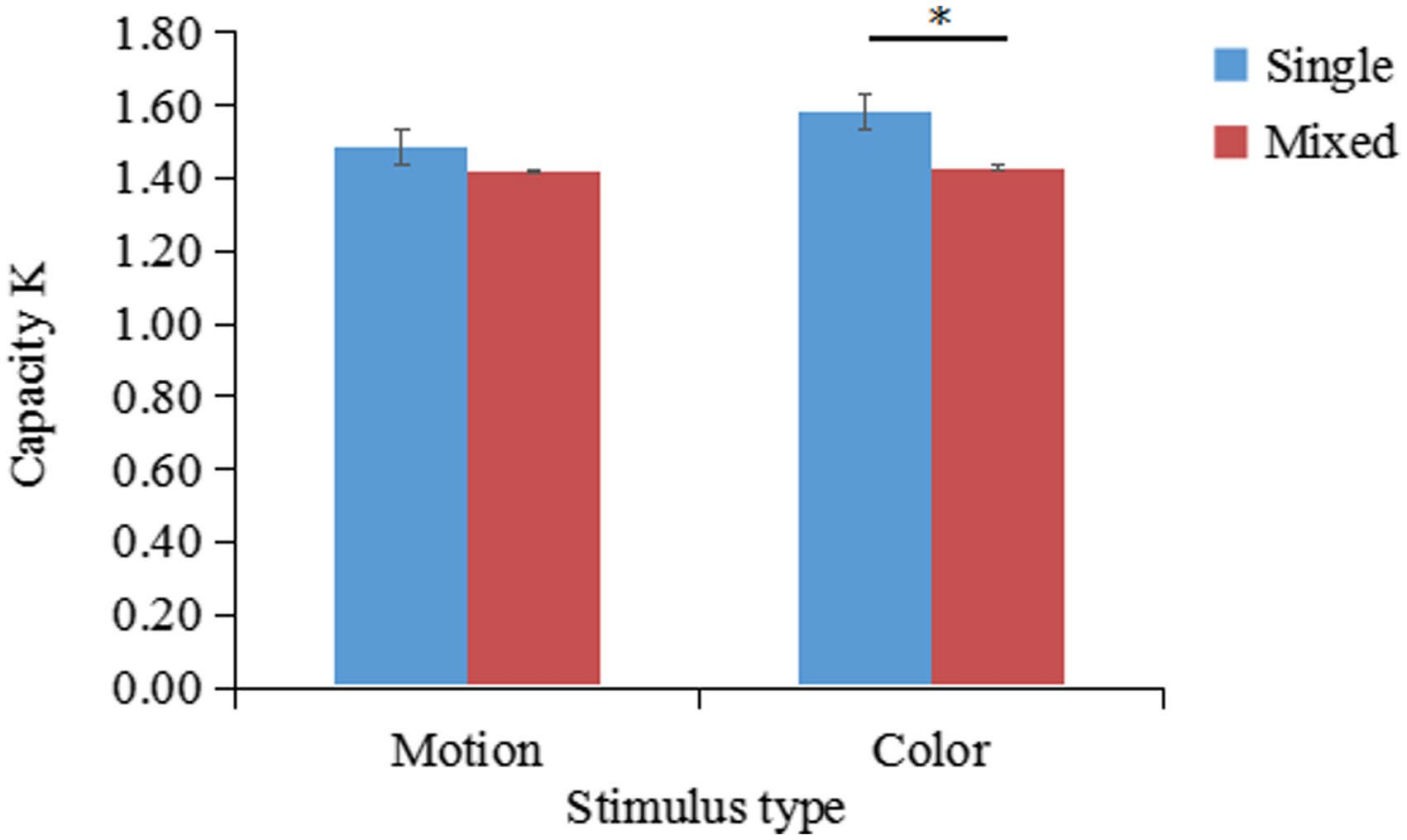
Changes in the working memory capacity for patterned movements (K) according to the stimulus and task type (results of Experiment 2a). *p < 0.05; **p < 0.01; ***p < 0.001.

The K values under different conditions are shown in Figure 5. A two-factor repeated-measures ANOVA on K indicated that the main effect of stimulus type was not significant $\left[F\left(1,31\right)=0.94,p=0.34,\eta_{p}^{2}=0.03\right]$; the main effect of task type was significant $\left[F\left(1,31\right)=5.70,p=0.02,\eta_{p}^{2}=0.16\right]$; the interaction between stimulus type and task type was not significant $\left[F\left(1,31\right)=0.92,p=0.35,\eta_{p}^{2}=0.03\right]$. Planned comparisons showed that K-single (M=1.58,SD=0.32) was significantly larger than K-mixed for color stimuli (M=1.43,SD=0.34), t(31)=2.13,p=0.04.

Experiment 2a revealed that the mean K-single/K-mixed ratio of remembering 2 stimuli (K-single) compared to remembering 4 stimuli (K-mixed) was 53% (SD = 0.17). A one-sample *t*-test suggested that the outcome of 53% was significantly lower than the outcome of 62% [t(31)=−2.12,p=0.001]. Therefore, patterned movement and color do not share the same storage buffer, indicating that patterned movement and color information are stored independently.

### 3.2. Experiment 2b: Storage of patterned movement and shape information

#### 3.2.1. Methods

##### 3.2.1.1. Participants

The sample size for Experiment 2b was calculated and participants were recruited in the same manner as in Experiment 2a. A new group of 31 students (15 females, aged 18–23 years, mean age: 21 ± 1.34 years) participated in Experiment 2b.

##### 3.2.1.2. Experimental design

Experiment 2b used 2 (stimulus type: motion and shape) × 2 (task type: single type and mixed type) within-subjects design. The stimulus types consisted of action stimuli or color stimuli. Each participant underwent 32 trials in each condition, preceded by 10 practice trials, for a total of 128 trials per participant. The order of the conditions was counterbalanced among participants.

##### 3.2.1.3. Experimental procedure

The knowledge items were shape or motion stimuli; otherwise, the experimental procedure of Experiment 2b was the same as that of Experiment 2a.

##### 3.2.1.4. Experimental stimuli

**Figure 6:**
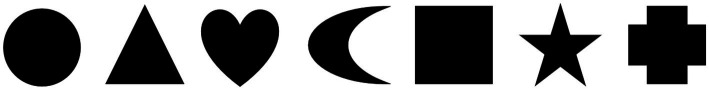
Shape stimuli for the experiment.

The motor stimulus was the same as in Experiment 2a, and the shape stimulus was as shown in Figure 6.

##### 3.2.1.5. Stimulus presentation

Stimulus presentation followed the same methods as in Experiment 1.

##### 3.2.1.6. Statistical analysis

First, the data with response times exceeding 3 standard deviations from the mean were excluded. Using the same Cowan formula as in Experiment 1 (Cowan, 2001), the working memory capacity of the participants in the different conditions was calculated. The data were tested for normality using the S–W test. The test results indicated that the data of this study conformed to the normal distribution (p>0.05). Then, we conducted a two-factor repeated-measures ANOVA on the working memory capacity K for stimulus type (motion and shape) × task type (single type and mixed type).

#### 3.2.2. Results

**Figure 7:**
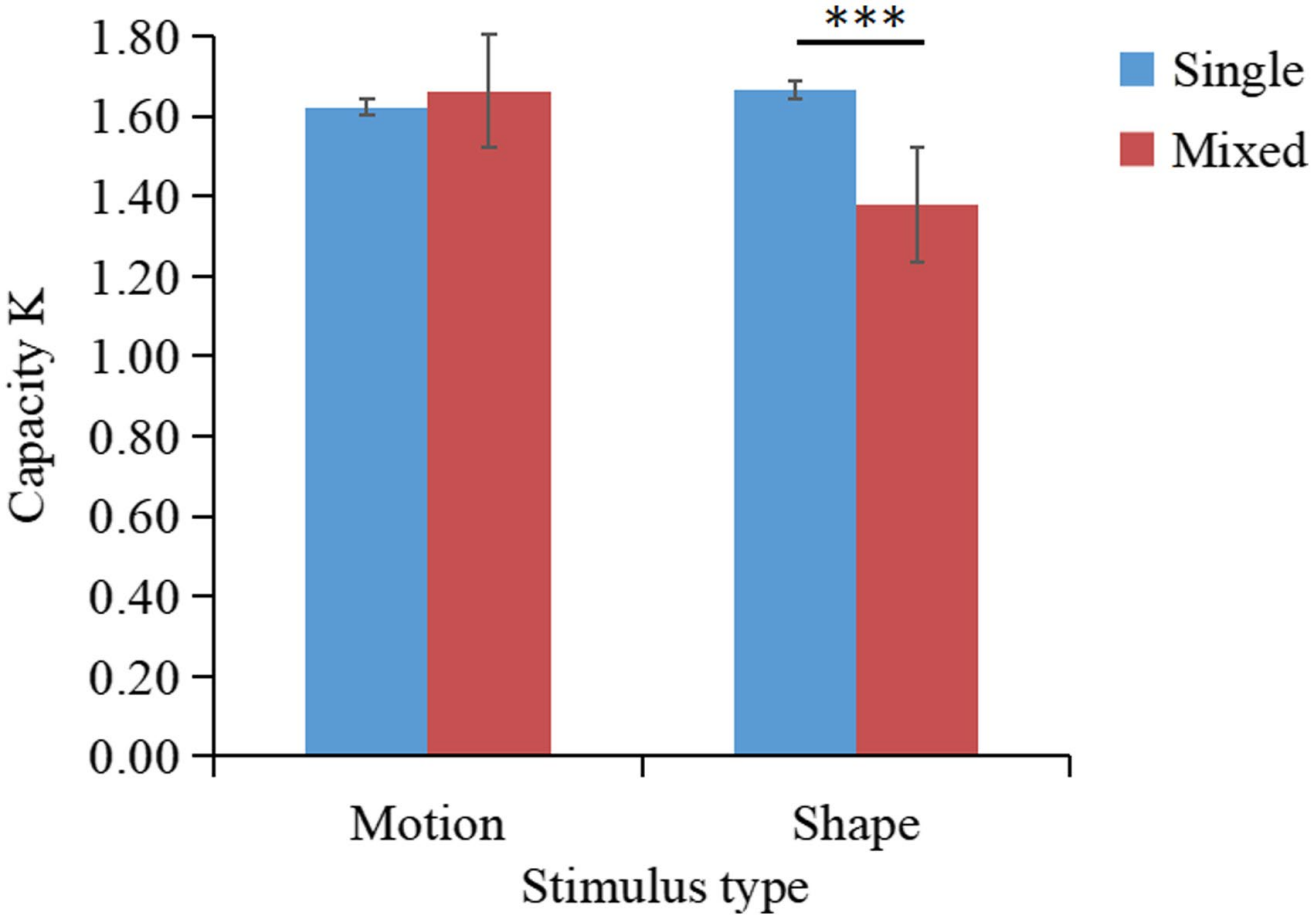
Changes in the working memory capacity for patterned movements (K) according to the stimulus type and task type (results of Experiment 2b). *p < 0.05; **p < 0.01; ***p < 0.001.

The K values of different conditions are shown in Figure 7. A two-factor repeated-measures ANOVA on K indicated that the main effect of stimulus type was significant $\left[F\left(1,30\right)=8.95,\;p=0.005,\;\eta_{p}^{2}=0.23\right]$; the main effect of task type was significant $\left[F\left(1,30\right)=6.83,\;p=0.01,\;\eta_{p}^{2}=0.19\right]$; the interaction between stimulus type and task type was significant $\left[F\left(1,30\right)=11.96,\;p=0.002,\;\eta_{p}^{2}=0.29\right]$. Simple effects analysis showed that K-single (M=1.66,SD=0.18) was significantly greater than K-mixed (M=1.38,SD=0.28) for shape stimuli, t(30)=5.04,p<0.001.

The mean K-single/K-mixed ratio for remembering 2 stimuli (K-single) compared to 4 stimuli (K-mixed) was 50% (SD = 0.14). A one-sample *t*-test revealed that the outcome of 50% was significantly lower than the outcome of 62% [t(30)=−4.83,p<0.001], indicating that motion and shape information do not share the same storage buffer. This result also indicated that the storage of motion information is independent of the visual subsystem.

## 4. Experiment 3: Association of patterned movements with spatial subsystems

### 4.1. Methods

#### 4.1.1. Participants

The sample size and participants for Experiment 3 were selected in the same manner as in Experiment 2. A new group of 31 students (18 females, aged 18–23 years, mean age 20.84 ± 1.12 years) participated in Experiment 3.

#### 4.1.2. Experimental design

Experiment 3 used a 2 (stimulus type: motion and location) by 2 (task type: single type and mixed type) within-subjects design. The stimulus types consisted of action stimuli or color stimuli. Each participant underwent 32 trials in each condition, preceded by 10 practice trials, for a total of 128 trials per participant. The order of the conditions was counterbalanced among participants.

#### 4.1.3. Experimental procedure

Location or motion stimuli were presented as probe items and memorized items. Otherwise, the experimental procedure of Experiment 3 was the same as that of Experiment 2.

#### 4.1.4. Experimental stimuli

**Figure 8:**
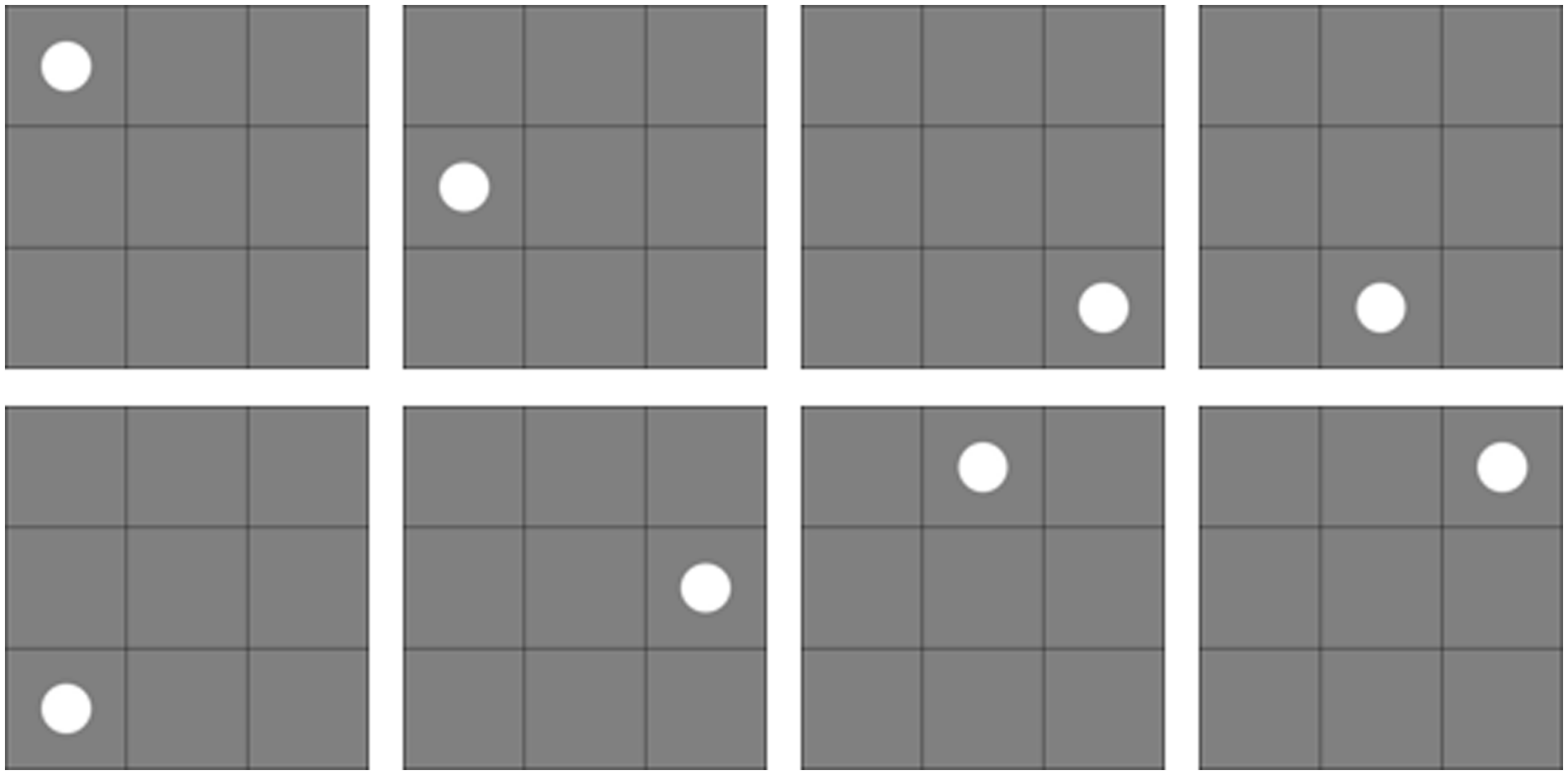
Location stimuli for the experiment.

A nine-box grid with 8 positions served as the location stimuli. Seven motion videos (the same as in Experiment 2) served as the motion stimuli. The location stimuli (graphs) were not repeated in a set of memorized items, and the size of each graph was approximately 3° × 3°. Location stimuli are shown in Figure 8.

#### 4.1.5. Stimulus presentation

Stimulus presentation followed the same approach as in Experiment 1.

#### 4.1.6. Statistical analysis

First, the data with response times exceeding 3 standard deviations from the mean were excluded. Using the same Cowan formula as in Experiment 1, the working memory capacity of the participants in the different conditions was calculated. The data were tested for normality using the S–W test. The test results indicated that the data of this study conformed to the normal distribution (p>0.05). Second, we conducted a two-factor repeated-measures ANOVA on the working memory capacity K for stimulus type (motion and location) × task type (single type and mixed type).

### 4.2. Results

**Figure 9:**
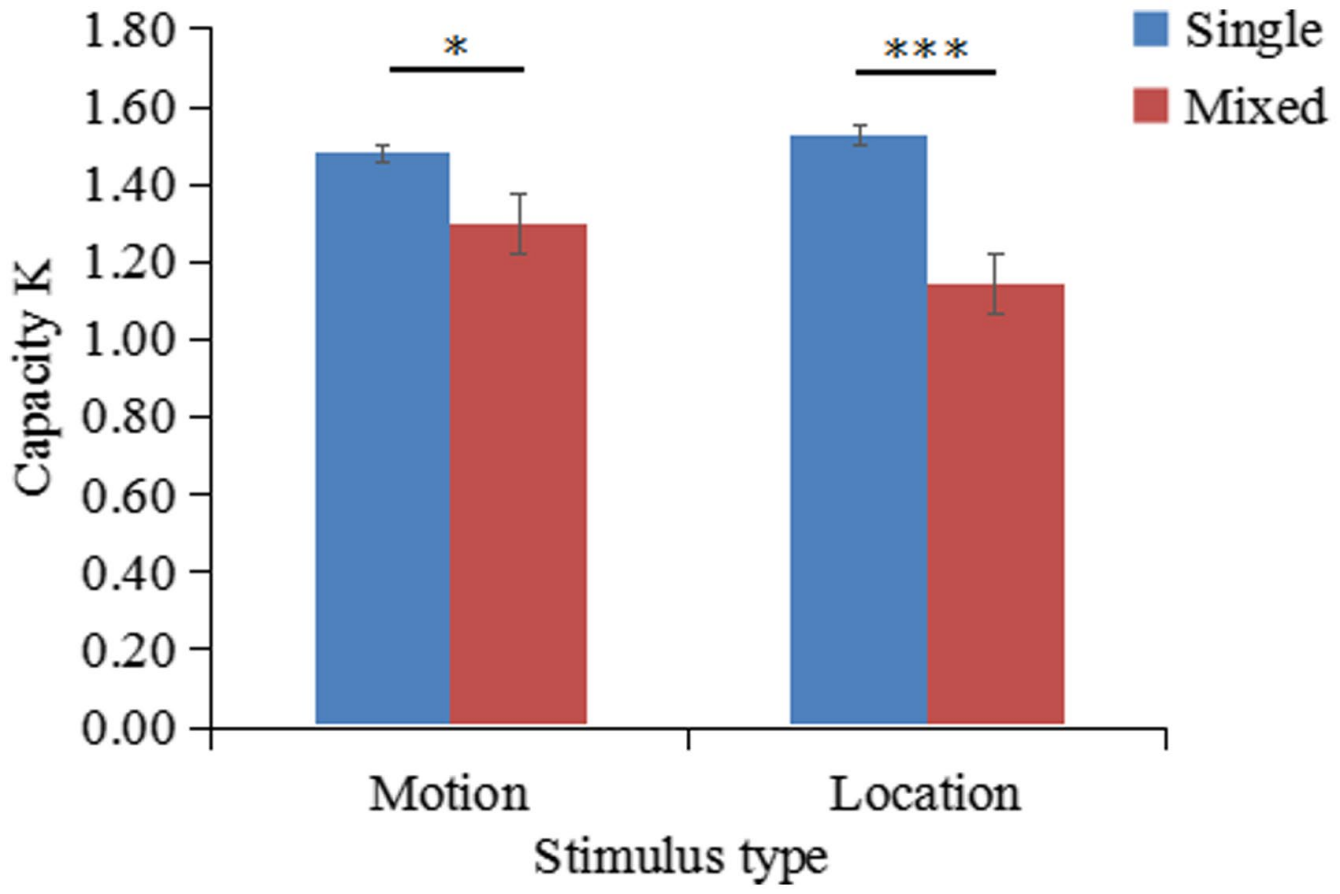
Changes in the working memory capacity for patterned movements (K) according to the stimulus type and task type (results of Experiment 3).

The K values of different conditions are shown in Figure 9. A two-factor repeated-measures ANOVA on K indicated that the main effect of stimulus type was not significant $\left[F\left(1,30\right)=0.87,\;p=0.36,\;\eta_{p}^{2}=0.03\right]$; the main effect of task type was significant $\left[F\left(1,30\right)=29.53,\;p<0.001,\;\eta_{p}^{2}=0.50\right]$; the interaction between stimulus type and task type was significant $\left[F\left(1,30\right)=4.14,\;p=0.05,\;\eta_{p}^{2}=0.12\right]$. Simple effects analysis showed that K-single (M=1.48,SD=0.35) was significantly greater than K-mixed (M=1.29,SD=0.36) for motion stimuli, t(30)=2.17,p=0.04. Additionally, K-single (M=1.53,SD=0.39) was significantly greater than K-mixed (M=1.14,SD=0.37) for location stimuli, t(30)=6.49,p<0.001.

The mean K-single/K-mixed ratio of remembering 2 patterned movements (K-single) or 4 patterned movements (K-mixed) was 63% (SD = 0.31). A one-sample *t*-test revealed that the difference between the outcome of 63 and 62% was not significant [t(30)=0.14,p=0.89].. However, the outcome of 63% was significantly higher than the outcome of 50% [t(30)=2.32,p=0.03]

The results of this experiment show that patterned movement and location information share the same storage buffer and that the storage of patterned movement information depends on the location subsystem.

## 5. Discussion

The present study explored the working memory capacity for patterned movements among various stimulus types and investigated the relationships of patterned movements with the visual and spatial subsystems of the visuospatial sketchpad. Three main findings were obtained. First, the working memory capacity for patterned movements significantly differed according to stimulus type. Specifically, the working memory capacity of the PLD sequences was significantly larger than that of the video stimuli. As the set size increased, the significant differences among the 3 different stimulus types reflected that changes in the stimulus type and difficulty of the task exerted different effects on the working memory capacity of participants. Changes in the stimulus type or task difficulty decreased the speed and efficiency of working memory processing for these participants and decreased recall. Second, the visual working memory and working memory of patterned movements are independent, indicating that the storage of patterned movement information is independent of the visual subsystem. Third, patterned movement information and spatial information share the same storage buffer, indicating that the storage of patterned movement information requires the spatial subsystem.

In Experiment 1, as the set size increased from two to five, participants’ response times increased and their accuracy decreased under different stimulus types. As the increase in the set size required individuals to focus more on task performance, their mental load increased correspondingly. The stimulus type is closely related to people’s existing knowledge and experience and exerts an effect on working memory, thus impacting the efficiency of task completion (Sel et al., 2014; Barley et al., 2021). The results of experiment 1 found that the difference in stimulus types had different effects on the size of the short-term memory capacity of individuals. Participants had the largest working memory capacity in the PLD stimulus condition and the smallest working memory capacity in the video stimulus condition. There was no significant difference between the 3D animations and the other two stimulus conditions. To complete the task, participants needed not only to encode the stimuli in a timely and accurate manner but also to store, refresh, and recall the encoded information in a timely and accurate manner to reach a final judgment and provide a keystroke response (Calvo-Merino et al., 2005; Brady et al., 2016).

The entire task, from the appearance of stimuli to the production of keystroke responses, involved a series of cognitive processes, such as perception, recognition, encoding, storage, updating, extraction, and decision-making (Guitard and Cowan, 2022a,b; Li and Cowan, 2022). All of these have high demands regarding attention (pointing and concentration), memory (short-term/working memory), and cognition (decision-making). The variation in the content and characteristics of the stimuli (with and without motion information) not only increased the difficulty of the task but also induced psychological stress (Galvez-Pol et al., 2018). They need to adjust their response rules and methods promptly, as well as to adapt to the changing stimuli and task requirements actively and quickly. By the “reaction time-accuracy trade-off,” individuals had to make decisions about the speed and accuracy of task completion based on task difficulty and task requirements to obtain the best possible performance (Galvez-Pol et al., 2018). With increases in set size, significant differences among the three stimulus types were revealed regarding reaction times, which are related to working memory capacity. This result suggested that the changes in stimulus type and task difficulty exerted different effects on the working memory capacity of participants for patterned movements. Changes in the stimulus type or difficulty led to decreased speed and efficiency of working memory processing, which hurts working memory capacity and decision-making.

In addition, Experiment 1 data were analyzed in terms of stimulus type. The accuracy of participants for PLD sequences was significantly higher than that for 3D animations and videos under different set sizes. This may be related to the different effects and roles of phonological loops (PLD sequences are easily encoded as having phonological meaning) and visuospatial templates (3D animations and videos are biased toward morphological meaning) in working memory. The results showed that the accuracy for videos was significantly lower than that for PLD sequences. This may be since the video stimuli are uncommon movements in daily life, which are unfamiliar to college students and not easily encoded verbally. In contrast, the PLD stimulus materials are common movements and behaviors in daily life, such as drinking and jumping. These movements are more familiar to college students, and they can be easily converted into figurative nouns, which are easier for individuals to remember and extract. Barley et al. (2021) identified picture bias in the picture-text source test. In other words, textual information was easily converted into picture information, which might transform the original bottom-up processing into top-down processing. This transformation consumed more cognitive resources (Barley et al., 2021). In contrast, participants in the present experiment were prone to the linguistic encoding of the PLD sequences, which led to bottom-up processing and did not consume many cognitive resources.

Experiment 2 revealed that when memorizing patterned movement, simultaneous memorization of color (Experiment 2a) or shape (Experiment 2b) information did not affect the memory of patterned movements. This finding suggested that patterned movement information has an independent storage buffer in working memory compared to visual information such as color and shape. A direct comparison between the single-type and mixed-type tasks verified whether patterned movements had independent storage space in working memory. When simultaneously remembering patterned movements and visual information such as color or shape, performance was not affected, and the K-single/K-mixed ratio was close to 50%, verifying the suggestion by Wood (2007) and Shen et al. (2014) that visual information, such as color and shape, are stored independently of patterned movement information in working memory (Wood, 2007, 2009, 2011; Poom, 2012; Shen et al., 2014). In addition, these findings validate the results of relevant brain imaging studies that provided evidence supporting the storage of motion information distinct from the visual subsystem, for example, remembering characteristic information about faces activated brain regions, such as the fusiform, parahippocampal, and inferior frontal gyri (Ungerleider, 1998). However, these brain regions were not activated during the motion memory task, suggesting that the neural basis of motion working memory differs from that of visual working memory.

In contrast, in Experiment 3, when participants simultaneously memorized motion and location information, the K-single/K-mixed ratio approached 62%. This finding suggested that the storage of patterned movement information requires the involvement of the spatial subsystem. The results of the present experiment are inconsistent with the findings of Shen et al. (2014) who used PLD sequences as experimental stimuli and concluded that spatial information and patterned movements are stored separately and independently in working memory. There are two possible reasons for this inconsistency. First, the experimental designs between Shen et al. (2014) study and the present study differed. In Shen et al. (2014) study, a compartmentalized design was used, with each type of condition corresponding to a compartmentalized group. Participants knew the memory task for each trial in this compartmentalized group. Moreover, in Shen et al. (2014) experiment, the spatial memory task in advance involved dots appearing on an invisible circle on the screen, whereas, in the present experiment, the dots appeared in a visible 9-box grid. A slight increase in task difficulty may have affected the experimental results. Second, in the present study, the experimental stimuli differed from those in studies supporting the independent storage of motion information relative to the spatial subsystem (Vicary and Stevens, 2014; Ding et al., 2015). The present study used 3D animations rather than simple PLD sequences. Both stimulus types require the individual to remember information about body configurations. However, the complexity of the two types differs in that the PLD sequences depict simple motions (Zihl and Heywood, 2015), unlike complex human motions (which are difficult to verbally encode). Future studies need to directly compare these two types of movements (simple vs. complex) and investigate whether the neural basis of both is the same, enhancing the understanding of the processing of patterned movement.

## 6. Conclusion

The present study provided behavioral evidence of the relationship between patterned movement information and the visuospatial sketchpad. We first investigated the effect of stimulus type on the working memory capacity for patterned movement using 3 different stimulus types. We assessed 3 metrics: accuracy, RT, and working memory capacity. Increases in set size revealed significant differences among the 3 stimulus types. Changes in the stimulus type or an increase in difficulty led to decreased speed and efficiency of working memory processing, negatively impacting decision-making.

We then conducted Experiments 2 and 3 to further explore the relationship between the storage of patterned movement information and the visuospatial sketchpad. First, we adopted the experimental paradigm of Shen et al. (2014) to collect behavioral data on patterned movement information as well as visual and spatial information. Specifically, our study examined the working memory capacities for patterned movement information, visual information, and spatial information as well as the relationship between patterned movement information and the visual and spatial subsystems. Our results showed that there was no interaction between visual working memory and working memory of patterned movements. This finding indicated that the storage of patterned movement information is independent of the visual subsystem. Second, we examined the relationship between spatial working memory and working memory of patterned movements. We separately assessed the working memory capacity for patterned movements and spatial working memory. The behavioral results indicated that the simultaneous use of spatial working memory and working memory of patterned movements affects the working memory of patterned movements. This finding suggested that patterned movement information and spatial subsystems may share cognitive resources.

Future research is needed to describe the functional interactions between the encoding and retrieval of patterned movement information. In addition, quantitative comparisons are needed as well as qualitative comparisons of single and mixed tasks are needed. Such studies will enhance our understanding of the neuroimaging characteristics of independent systems of visual working memory and spatial working memory, as well as the storage mechanisms underlying visuospatial working memory and motion working memory.

## Data availability statement

The raw data supporting the conclusions of this article will be made available by the authors, without undue reservation.

## Ethics statement

Ethical review and approval was not required for the study on human participants in accordance with the local legislation and institutional requirements. The patients/participants provided their written informed consent to participate in this study.

## Author contributions

CL wrote the original draft. CL, YH, and CW contributed to the design of the study. WT translated and polished the first draft. XX and YL conducted part of the experiment. CL, WT, and LB performed the statistical analysis. YL and TX gave valuable comments on the revision of the experimental material. XL and SW contributed to the manuscript revision. All authors have read and agreed to the submitted version of the manuscript.

## Funding

This work was supported by Research on Major Issues in Aviation Medicine (2019ZTD04), Air Force Recruiting Bureau Key Programs (KJZFJ2020-3), Key Projects of the Basic Strengthening Program of the Science and Technology Commission of the Military Commission (2020-JCJQ-ZD-263-04), Air Force Equipment Intramilitary Research Program Key Projects (KJ20182A030138), and Key Projects of the 14th Five-Year Plan of Army Logistics Research Program (BKJ19J021).

## Conflict of interest

The authors declare that the research was conducted in the absence of any commercial or financial relationships that could be construed as a potential conflict of interest.

## Publisher’s note

All claims expressed in this article are solely those of the authors and do not necessarily represent those of their affiliated organizations, or those of the publisher, the editors and the reviewers. Any product that may be evaluated in this article, or claim that may be made by its manufacturer, is not guaranteed or endorsed by the publisher.

## Abbreviations

PM: patterned movements
WMC: working memory capacity
PLD: point-light display
